# Chicago Medical Society

**Published:** 1881-07

**Authors:** 


### ARTICLE VIII.

### CHICAGO MEDICAL SOCIETY.

Stated meeting, May 15, 1881. Dr. E. Ingals, President, in the chair.

The paper of the evening, on

NUMERATION AND MEASUREMENT, was read by Dr. Weller. He said that the idea upon which the Decimal System was founded was as old as written history; a

Roman scale, constructed as a lever with unequal arms, and graduated in decimals, had been exhumed from the ruins of Pompeii, while unmistakable evidences were found that the decimal scale was applied to measures of length and weight in Egypt in the days of the Pharaohs, in Chaldea, and when the Sanscrit was the popular language of the Hindoos. Ingenious mathematicians have constructed systems based on different radices—four, six, eight, twelve, sixteen, twenty-four and sixty-four. Charles XII of Sweden, constructed one based on twelve. Emmanuel Swedenborg, one on sixty four; but, "man, to whose nature imperfection clings as a part of it, cannot think a perfect thought, much less construct a perfect system."

He now proposed a system based on the radix eight, which Dr. Taylor, of Philadelphia had discussed before the Pharmaceutical Association in 1859. This new system needed new characters and a new nomenclature. The first eight numbers would be *un*, *du*, *te*, *fo*, *pa*, *si*, *he*, *ok*. Zero would become *ze*. *Unok* or *nok* would be nine; *nokun*, eleven; *dok*, sixteen, *fok*, thirty-two, etc. A letter added to and preceding one of the numbers becomes a multiplicator, placed after it, it means addition. He did not believe the unit of the decimal system had any advantage over other measures of length, and said that the avoirdupois pound should be retained in this country; but this he proposed to divide according to his new system.

Followed a discussion of the paper by Drs. Whitmire and Ch. Kruzemark, the latter had adopted the decimal system and was well pleased with it.

### MATERNAL IMPRESSIONS.

Dr. H. D. Valin reported a case of that nature. He had lately delivered a woman of a big boy, and found the child's nose flattened on the right side. The mother said that when she was about three and a half months pregnant, she had entered a meat market where she met a man whose nose had been eaten away by a cancer. It made a strong impression upon her, and there was no doubt in her mind as to its having caused the child's nose to assume such deformity.

Dr. Weller had met a wonderful case of that nature. When three and a half months pregnant, a woman had fallen down a ladder, and after delivery at full term, it was found that the baby's right leg had been broken below the knee, and that the fragments of the bones had united with a good deal of deformity.

Dr. J. S. Whitmire had seen a case of congenital fracture of the femur, following a tedious labor, but he had not been able to ascertain how the bone had been broken.

Dr. Weller related another case of maternal impression, in which the mother asked him before she had seen her baby, whether it had a white spot on the left eye? She had discovered an artificial eye from the manner in which the light was reflected from it, when in her third month of pregnancy. The child had such a mark and has it yet. He is an employè of the North-Western Railway Co.

Dr. R. A. Leonard said he had in his museum a fœtus whose features resemble very much those of a beggar on State street who had come under the mother's observation. Fœtus was acephalic and remarkable in many respects.

Dr. Jas. E. Mead was elected a member, and the name of W. H. Curtis proposed.

---

ANNUAL death rate per 1,000 in the chief cities of the United States for the year 1880 :

| | Population. | Total No. of deaths. | Annual death-rate per 1000. |
|---|---|---|---|
| Boston | 362,535 | 8,634 | 23.5 |
| Providence | 104,850 | 2,098 | 20 |
| New York | 1,206,577 | 32,245 | 26.7 |
| Brooklyn | 566,689 | 13,576 | 24 |
| Philadelphia | 846,980 | 17,701 | 20.9 |
| Baltimore | 332,190 | 8.216 | 24.7 |
| District of Columbia | 180,000 | 4,122 | 22.9 |
| New Orleans | 216,140 | 5,631 | 24.2 |
| Louisville | 123,625 | 2,618 | 21.2 |
| Cincinnati | 255,708 | 5,331 | 20.9 |
| Cleveland | 160,140 | 3,260 | 20.4 |
| Chicago | 503,304 | 10,469 | 20.8 |
| Milwaukee | 115,578 | 2,s91 | 21.5 |
| St. Louis | 350,522 | 6,725 | 19.2 |
| San Francisco | 233,956 | 4,518 | 19.3 |

—*National Board of Health Bulletin.*

